# Prevalence, Comorbidity and Heritability of Hoarding Symptoms in Adolescence: A Population Based Twin Study in 15-Year Olds

**DOI:** 10.1371/journal.pone.0069140

**Published:** 2013-07-10

**Authors:** Volen Z. Ivanov, David Mataix-Cols, Eva Serlachius, Paul Lichtenstein, Henrik Anckarsäter, Zheng Chang, Clara Hellner Gumpert, Sebastian Lundström, Niklas Långström, Christian Rück

**Affiliations:** 1 Department of Clinical Neuroscience, Karolinska Institutet, Stockholm, Sweden; 2 Department of Medical Epidemiology and Biostatistics, Karolinska Institutet, Stockholm, Sweden; 3 Departments of Psychosis Studies and Psychology, King’s College London, Institute of Psychiatry, London, England; 4 Centre for Ethics Law and Mental Health, University of Gothenburg, Gothenburg, Sweden; 5 Gillberg Neuropsychiatry Centre, Institution of Neuroscience and Physiology, University of Gothenburg, Gothenburg, Sweden; University of Hong Kong, Hong Kong

## Abstract

**Background:**

Hoarding Disorder (HD) is often assumed to be an ‘old age’ problem, but many individuals diagnosed with HD retrospectively report first experiencing symptoms in childhood or adolescence. We examined the prevalence, comorbidity and etiology of hoarding symptoms in adolescence.

**Methods:**

To determine the presence of clinically significant hoarding symptoms, a population-based sample of 15-year old twins (N = 3,974) completed the Hoarding Rating Scale-Self Report. Co-occurring Obsessive Compulsive Disorder (OCD), Autism Spectrum Disorders (ASD) and Attention Deficit Hyperactivity Disorder (ADHD) were estimated from parental report. Model-fitting analyses divided hoarding symptom scores into additive genetic, shared, and non-shared environmental effects.

**Results:**

The prevalence of clinically significant hoarding symptoms was 2% (95% CI 1.6–2.5%), with a significantly higher prevalence in girls than boys. Exclusion of the clutter criterion (as adolescents do not have control over their environment) increased the prevalence rate to 3.7% (95% CI 3.1–4.3%). Excessive acquisition was reported by 30–40% among those with clinically significant hoarding symptoms. The prevalence of co-occurring OCD (2.9%), ASD (2.9%) and ADHD (10.0%) was comparable in hoarding and non-hoarding teenagers. Model-fitting analyses suggested that, in boys, additive genetic (32%; 95% CI 13–44%) and non-shared environmental effects accounted for most of the variance. In contrast, among girls, shared and non-shared environmental effects explained most of the variance, while additive genetic factors played a negligible role.

**Conclusions:**

Hoarding symptoms are relatively prevalent in adolescents, particularly in girls, and cause distress and/or impairment. Hoarding was rarely associated with other common neurodevelopmental disorders, supporting its DSM-5 status as an independent diagnosis. The relative importance of genetic and shared environmental factors for hoarding differed across sexes. The findings are suggestive of dynamic developmental genetic and environmental effects operating from adolescence onto adulthood.

## Introduction

Hoarding has been defined as the acquisition of and failure to discard a large number of possessions, difficulties using living spaces due to clutter and significant impairment and/or emotional distress due to the hoarding behavior [1]. Hoarding can be a serious health problem and may result in social isolation, family burden, eviction, and even death in extreme cases [2]. Although traditionally studied in the context of Obsessive-Compulsive Disorder (OCD), mounting research indicates that hoarding is in fact seldom OCD-related [3]. The idea that hoarding might be a separate psychopathological entity has led to the inclusion of a new disorder in DSM-5 named Hoarding Disorder (HD) [4]. The diagnostic criteria for HD have been empirically tested and found to be valid, reliable and perceived as useful and acceptable by both professionals and sufferers alike [5], [6].

Hoarding is often considered a problem of old age; most clinically referred samples consist of adults or older adults. However, anecdotal evidence and some retrospective studies suggest that the origins of hoarding may be in childhood or adolescence [7], [8]. Indeed, many patients report specific events in childhood (such as parents forcefully discarding possessions) as the origin of their current problematic behavior [9]. HD is thought to follow a chronic and progressively deteriorating course over time, with a retrospectively reported onset between ages 11 and 15 [8]. In adults, hoarding may be highly prevalent with estimates of point prevalence ranging from 2% to almost 6% [10], [11]. The actual prevalence of hoarding symptoms during childhood and adolescence in the general population remains unknown. Because parental control over the living space may limit the extent and consequences of hoarding in young people, any prevalence estimates should take this into account.

The most commonly reported psychiatric comorbidities of HD are Major Depressive Disorder, Generalized Anxiety Disorder, Social Phobia and Attention Deficit Hyperactivity Disorder (ADHD), whereas OCD is less frequently associated with hoarding [6], [12]. Several studies have examined the presence and correlates of hoarding symptoms among children and adolescents with OCD [13]–[15]. Hoarding in children with OCD is associated with lower insight and higher levels of externalizing disorders compared to children with OCD who do not hoard [16]. Hoarding symptoms have also been described in clinical samples of youth and adults with Autism Spectrum Disorder (ASD) [17] and ADHD [18]. However, it seems important to establish to what extent hoarding symptoms in childhood/adolescence are associated with these common neuropsychiatric disorders in the general population, free from the selection biases involved with clinically ascertained samples.

Research into the etiology of HD is still in its infancy. Uncontrolled clinical studies suggest that hoarding runs in families, [19]–[21] and twin studies indicate that approximately 50% of the liability to hoarding symptoms can be attributed to genetic factors whereas unique (or non-shared) environmental factors explain the remaining variance [10], [22], [23]. While the largest twin study was conducted in adult females [10], [22], Taylor et al. [23] found no evidence of gender differences in the heritability of hoarding. However, it is well known that heritability estimates of behavioral traits can change substantially with age, and that gender differences in heritability patterns may be present in adolescents but not in adults [24]–[27]. Thus, data on the heritability of hoarding symptoms in younger people could potentially increase our knowledge of the dynamic etiological factors that may be at play across the lifespan.

The first aim of this study was to elucidate prevalence and comorbidity of hoarding symptoms in a large twin sample of adolescents. The second aim was to estimate the relative contributions of genetic and environmental factors to hoarding symptoms and to test whether sex-differences in heritability patterns exist in this age group.

## Methods

### Participants

Participants were 15-year old monozygotic (MZ) or dizygotic (DZ) twins enrolled in the Swedish Twin Registry and taking part in the ongoing Child and Adolescent Twin Study in Sweden (CATSS). CATSS is a prospective, longitudinal study of all twins born in Sweden since 1992 whose parents were first contacted and interviewed when twins reached the age of 9 or 12 years [28]. We used data on hoarding symptoms from the follow-up at age 15, when twins themselves were contacted and asked to fill out a measure of hoarding symptoms. This age group is ideal to study the development of hoarding symptoms given the available retrospective accounts of adolescent symptom onset given by adult patients with HD [8], [29].

The current study sample is comprised of (1) twins who, according to parental interviews, screened negative for neurodevelopmental disorders at age 9/12 (n = 3,713) and (2) twins from the same cohort, who screened positive for a neurodevelopmental disorder and age-matched random healthy twins (n = 261). Twins from the latter group were invited to participate in a clinical face-to-face examination at age 15 and hoarding symptoms were captured with the same questionnaire but in connection to the clinical examination. Twin zygosity was determined either by using single nucleotide polymorphisms (SNPs) [30] or (if DNA was unavailable) an algorithm based on twin similarity with predictive value greater than 95% compared to DNA testing [31].

Ethical approval was obtained from the Regional Ethics Review Board in Stockholm under contracts Dnr 02–289, 2009–793 and 03–672. In accordance with the board’s decision and Swedish regulations, written informed consent was obtained directly from the 15-year olds involved in this study and not from their parents. However, parents were informed about the study and what was being collected. Parents provided written informed consent regarding their own participation in the parental interviews when twins were 9 and 12 years old.

A total of 3,974 participants (response rate = 47% of all Swedish twins in the age group) were included in the current study. Of these, 45% (n = 1,805) were boys and 55% (n = 2,169) girls. Furthermore, 30% (n = 1,210) were monozygotic (MZ) twins, 30% (n = 1,119) dizygotic same-sex (DZ), 26% (n = 1,022) dizygotic opposite sex (DZOS) twins and 14% (n = 543) had unknown zygosity. There were no significant differences between participants who responded to the hoarding questionnaire in the CATSS-battery compared to those who did not (n = 218) in terms of dwelling area (in both groups, 69% lived in a city rather than a rural area), having a mother born abroad (9% vs.10%, respectively), a father born abroad (8% vs. 11%), parental income, or paternal educational level. However, a larger proportion of mothers to responders had a university education compared to non-responders (47 vs. 39%, z = −2.12, df = 1, p<0.05). Co-occurring OCD at age 9/12 was also equally common in both groups (1% among responders vs. 3% among non-responders; χ2 = , 2.00, df = 1, *p* = 0.20), whereas more non-responders screened for ASD (2% vs. 6%; χ2 = 8.62, df = 1, *p*<0.01) and ADHD (7% versus 17%; χ2 = 22.28, df = 1, *p*<0.001). Further, a larger proportion of the responders were girls (55% vs. 45%; χ2 = 5.05, df = 1, *p*<0.05), and monozygotic (35% vs. 27%; χ2 = 4.71, df = 1, *p*<0.05) than non-responders.

### Measures

All study participants filled out the Hoarding Rating Scale-Self Report (HRS-SR) [32]. The HRS-SR consists of five items measured on a 9-point Likert type scale ranging from 0 (none) to 8 (extreme). The HRS-SR items reflect the proposed DSM-5 criteria for HD: clutter in the rooms of one’s home, difficulty discarding possessions, excessive acquisition and perceived distress and impairment. To address adolescents’ limited control over their entire homes and parental control, we rephrased the clutter item in the questionnaire to refer solely to clutter in the young person’s own room (rather than the entire home).

In the current study, the HRS-SR displayed acceptable internal consistency (Cronbach’s alpha = 0.70) and a principal component analysis revealed a single factor structure explaining 46.2% of the variance, with factor loadings ranging from 0.65 (clutter in room) to 0.71 (emotional distress).

Presence of clinically significant hoarding symptoms was defined as scoring at least moderately (4 or more out of 8) on items measuring clutter, difficulties discarding and distress and/or impairment. This has previously been described as criteria for clinically significant hoarding [33], [34] and approximately corresponds to the proposed core criteria for DSM-5 [3]. Although it is currently unclear whether the DSM-5 diagnostic criteria are suitable for adolescents [35], a recent taxometric exploration of the latent structure of hoarding symptoms [36] supported a similar dimensional structure of hoarding in adults and young adults (mean age = 19). However, since the phenomenology of hoarding in adolescence is largely unknown, we also calculated an additional prevalence estimate. For this, we used the above-described criteria but excluded the clutter criterion since clutter, or the lack thereof, could be heavily influenced by familial factors, such as parents or siblings discarding items. Additionally, since excessive acquisition is a prominent feature of hoarding (but not a core criterion in DSM-5) we also calculated the proportion of participants reporting excessive acquisition among those endorsing clinically significant hoarding symptoms.

Co-occurring neurodevelopmental disorders were assessed at age 9/12 with the ‘Autism-Tics, AD/HD and other Comorbidities inventory’ (A-TAC) [37], [38] specifically developed for parental interviewing in CATSS. Parents to twins underwent telephone interviews answering questions regarding lifetime symptoms of ADHD and ASD closely mapped after the corresponding DSM-IV-TR-criteria [39]. The A-TAC is structured in different modules, which in turn consist of several items: *Language* (6 items), *Social interaction* (6 items), and *Flexibility* (5 items) for ASD and *Concentration/Attention* (9 items) and *Impulsiveness/Activity* (10 items) for ADHD. In each module, parents are first reminded that the items refer to a life-time perspective and subsequently asked to answer either by stating *No* (Score 0), *Yes, to some extent* (Score 0.5) or *Yes* (Score 1). Additionally, parents are given the alternatives *Do not know* or *Do not wish to answer*. To be defined as screening positive for ASD in this study, a cut-off score of 4.5 was required. Conversely, for ADHD, a cut-off score of 6 was used. These cut-offs have been validated and provide high sensitivity/specificity (0.91/0.80) for ASD and (0.91/0.73) ADHD as well as excellent inter-rater agreement (ICC>0.90) [38]. Furthermore, predictive validity, measured by receiver operating characteristics (ROC) is excellent with areas under the curve (AUC) of 0.96 for ASD and 0.94 for ADHD. OCD symptoms were ascertained with two A-TAC items, modeled after DSM-IV-TR-criteria for OCD, addressing obsessions and compulsions. Examples of typical obsessions concerning dirt, contamination or catastrophes and compulsions consisting of washing, touching, repeating, arranging or counting were provided. The cut-off score for OCD was determined to 1.

### Statistical Analyses

Internal consistency and factor analysis of the HRS-SR as well as prevalence estimates and demographic differences were estimated using SAS version 9.2 [40]. For continuous variables we used *t-*tests, whereas ordinal and nominal data were analyzed with χ2-tests (Fischer’s exact test) and Wilcoxon’s test. In the prevalence estimates, we controlled for the clustering of twins within families. All parameter estimates in the twin analyses were obtained from maximum-likelihood estimates in MX [41].

### Twin Analyses

Twin analyses are based on the differing genetic relatedness of MZ twins, sharing all of their genes and DZ twins, which share on average 50% of their co-segregating genes. The assumption that both twin types grow up under equal environments leads to the conclusion that an accentuated resemblance within MZ compared to DZ pairs on a given trait is due to a genetic influence on that trait. To estimate the magnitude of this within-twin pair resemblance, we calculated intraclass correlations for HRS-SR total scores by zygosity. Since total scores were positively skewed (skewness = 1.55), they were successfully logarithmically transformed using the natural logarithm (skewness = −0.14). Next, we undertook maximum-likelihood univariate model-fitting [42] and fitted our raw data to different models to identify that with the best fit by comparing the chi-square statistic of the −2 Log likelihood (−2LL) statistics. Twin models decompose the variance of phenotypes to additive genetic factors (A), shared environmental factors (C; which make twins within a pair alike), and non-shared environmental factors (E; which make twins within a pair dissimilar). The correlational pattern showed that the MZ twins were more alike compared to DZ twins among males but not among females, suggesting possible sex differences in genetic and environmental influences on hoarding symptoms. Therefore we fitted several sex-limitation models to our data. Initially, however, data was fitted to the saturated model where variances and means of the first and second born MZ and DZ twin were allowed to vary freely. Next, we fitted data to the General Sex Limitation Model (GSM) [42] which allows for qualitative and quantitative sex effects on a trait (i.e., we tested whether the same genes influenced hoarding symptoms in both sexes and if the magnitude of this genetic effect was equal across sexes). Additionally, the genetic correlation between DZOS twins was allowed to vary freely and so were the effects of A, C and E on males and females. We also tested a similar model where the shared environmental correlation in DZOS twins was allowed to vary freely to test if qualitative differences in the shared environment could be influential across sexes. In striving for parsimony by using as few parameters in a model as possible, we subsequently tested the more restrained Common Effects Sex Limitation Model (CESM) [42] and compared this to the full GSM. In CESM only the magnitude of the genetic effect is allowed to vary whereas the genes affecting the trait are common to both sexes by restraining the DZOS genetic correlation to 0.5. Finally, we also tested a No Sex Effects Model (NESM) [42] in which A, C and E effects are all restrained to be equally influential on both sexes and compared this to the CESM.

## Results

### Prevalence

A total of 2% (N = 79; 95% CI 1.6%−2.5%; Mean HRS-SR score = 19.7; min = 12, max = 40; SD = 4.9) of the entire sample of 15-year olds had clinically significant hoarding symptomatology based on the above-described criteria. The prevalence was significantly higher in girls (2.6%; 95% CI 2.0%−3.5%) than in boys (1.2%; 95% CI 0.8%−1.8%) (χ2 = 15.5, df = 1, *p*<0.01). After excluding the clutter criterion, the prevalence rate increased to 3.7% (N = 145; 95% CI 3.1%−4.3%; Mean HRS-SR score = 17.0; min = 8, max = 40; SD = 5.3).

Excessive acquisition was endorsed by 38% (n = 30) of participants with clinically significant hoarding symptoms (including clutter) and by 28% (n = 41) of participants when the clutter criterion was excluded from the prevalence estimates.

### Demographic Characteristics and Comorbidity

Socio-demographic and clinical characteristics of hoarding and non-hoarding groups are shown in Table 1. Adolescents with hoarding symptoms were more likely to be female (72% vs. 54%). The two groups were similar regarding urban area of living, parental education, income and immigrant status. Among hoarders, ADHD was the most common co-occurring disorder at age 9/12 (10.0%) whereas OCD and ASD were each comorbid in 2.9% of the cases. However, no significant differences emerged between the hoarding and the non-hoarding groups in terms of comorbidity.

**Table 1:** Characteristics of 15-year old twins in Sweden fulfilling criteria for clinically significant hoarding symptoms according to the HRS-SR and those who did not.

	Hoarding (N = 79)	Non-hoarding (N = 3,895)	t	Wilcoxon’s Z	?2	*p*
Female sex, n (%)	57 (72.2)	2112 (54.2)	–	–	10.04	<0.01
Monozygotic, n (%)	22 (31.9)	1188 (35.3)	–	–	0.35	0.61
Living in urban area, n (%)	52 (74.3) (missing = 9)	2337 (69.3) (missing = 522)	–	–	0.81	0.43
Father’s highest education, n (%)			–	0.73	–	0.47
Primary school	13 (16.5)	554 (14.2)				
High School	30 (38.0)	1852 (47.5)				
University <3 yrs	10 (12.7)	391 (10.0)				
University ≥3 yrs	25 (31.7)	1022 (26.2)				
PhD	1 (1.3)	72 (1.9) (missing = 4)				
Mother’s highest education, n (%)			–	0.83	–	0.41
Primary school	5 (6.3)	145 (6.3)				
High School	39 (49.4)	1768 (45.4)				
University <3 yrs	6 (7.6)	191 (4.9)				
University ≥3 yrs	28 (35.4)	1661 (42.6)				
PhD	1 (1.3)	28 (0.7) (missing = 4)				
Mean annual income father, SEK (SD)	361,7 00 (541,700)	346,100 (351,400)	–0.38	–	–	0.70
Mean annual income mother, SEK (SD)	245,000 (92,100)	262,400 (150,600)	1.02	–	–	0.31
Father born abroad, n (%)	7 (11.3) (missing = 17)	225 (7.5) (missing = 898)	–	–	1.24	0.23
Mother born abroad, n (%)	7 (10.3) (missing = 11)	304 (9.2) (missing = 572)	–	–	0.10	0.67
OCDa, n (%)	2 (2.9) (missing = 9)	49 (1.5) (missing = 517)	–	–	0.93	0.28
ADHDa, n (%)	7 (10.0) (missing = 9)	244 (7.2) (missing = 520)	–	–	0.78	0.38
ASDa, n (%)	2 (2.9) (missing = 9)	74 (2.2)	–	–	0.14	0.67

**Note**: SEK = Swedish Crowns, OCD = Obsessive Compulsive Disorder, ADHD = Attention Deficit Hyperactivity Disorder. ASD = Autism Spectrum Disorders. a) Determined from parental reports at age 9/12 years.

### Heritability

In total, 1555 twin pairs were included in the model-fitting analyses. The intraclass correlations for HRS-SR hoarding symptoms are provided in Table 2. In MZ boys, the correlation (0.44; 95% CI 0.33–54) was significantly higher than the DZ correlation (0.17; 95% CI 0.06–0.29), indicating that genes had an influence on hoarding among males. In contrast, we found no significant difference in MZ (0.35; 95% CI 0.25–0.44) and DZ (0.41; 95% CI 0.31–0.51) correlations among girls, suggesting that familial similarity regarding could be explained by shared environment rather than genes.

**Table 2:** Intraclass correlations for hoarding symptoms according to the HRS-SR in Swedish twins at age 15 years.

	N (pairs)	ICC	95% CI
MZ males	231	0.44	0.33–0.54
DZ males	279	0.17	0.06–0.29
MZ females	336	0.35	0.25–0.44
DZ females	265	0.41	0.31–0.51
DZOS	444	0.16	0.07–0.25

**Note:** MZ = Monozygotic twins; DZ = Dizygotic same sex twins; DZOS = Dizygotic opposite-sex twins.

Table 3 provides model-fitting results. Compared to the initial saturated model, the GSM did not provide a significantly worse fit (*p* = 0.88). Similarly, testing the more parsimonious CESM did not result in a worsening of fit compared to the GSM (*p* = 0.99), suggesting no qualitative genetic/shared environmental sex differences (i.e., the same genes/shared environments are important for boys and girls). However, in comparison to the CESM the NESM, in which genetic and environmental influences are equated across sexes, resulted in a significant deterioration of the fit (*p* = 0.02). Thus, the model with the best fit was the CESM, indicating that the same genes/shared environments accounted for the variation in hoarding symptoms in both adolescent males and females, but that the magnitude of this influence varied across sexes.

**Table 3:** Model-fitting results for hoarding symptoms in Swedish twins at age 15 years.

Model	−2LL	?2	Δ df	*p*	AIC	Compared to model
1. Saturated Model	8182.713				2012.71	
2. General Sex Limitation Model*	8192.436	9.72	16	0.88	1990.44	1
3. General Sex Limitation Model**	8192.436	9.72	16	0.88	1990.44	1
**4. Common Effects Sex Limitation Model**	**8192.436**	**0**	**1**	**0.99**	**1988.44**	**2, 3**
5. No Effects Sex Limitation Model	8201.754	9.318	3	0.02	1991.754	4

**Note:**

The General Sex Limitation Model tested for qualitative and quantitative sex differences.

* Testing for the presence of qualitative genetic effects across sexes.

** Testing for the presence of qualitative shared environmental effects across sexes.

The Common Effects Sex Limitation Model tested for quantitative sex differences and the No Effects Sex Limitation Model tested for no differences across sexes.

The best fitting model is bolded.

−2LL = minus twice the log likelihood; χ2 = differences in −2LL statistic between submodel and full model; Δ df = change in degrees of freedom between submodel and full model; *p = *probability; AIC = Akaike Information Criterion.

Table 4 summarizes the standardized parameter estimates of the best-fitting model (i.e. the CESM). The figures indicated that in boys, genes accounted for 32% (95% CI 13–44%) of the phenotypic variance in hoarding symptoms whereas shared environmental factors (4%; 95% CI 0–17%) only had a negligible effect. Non-shared environmental effects and measurement error (64%; 95% CI 55–75%) accounted for the largest proportion of the variance. By contrast, genes only accounted for a non-significant 2% (95% CI 0–24%) of the variance in girls, whereas the shared environment (32%; 95% CI 14–41%) and the non-shared environment plus measurement error (65%; 95% CI 58–73%) accounted for most of the phenotypic variance.

**Table 4:** Explained variance by additive genetic and environmental factors to hoarding symptoms in Swedish twins at age 15 years according to best fitting model.

	A (95% CI)	C (95% CI)	E (95% CI)
Males	0.32 (.13–44)	0.04 (.00–17)	0.64 (.55–75)
Females	0.02 (.00–24)	0.32 (.14–41)	0.65 (.58–73)

**Note:** A = additive genetic effects; C = shared environmental effects; E = non-shared environmental effects.

## Discussion

As proposed by retrospective studies of adults [8], [9] and studies of children with OCD exhibiting hoarding symptoms [13]–[15] our study suggests that hoarding symptoms are relatively prevalent (approximately 2%) among adolescents although somewhat less prevalent than in similar adult studies [11], [43]. Mean scores on HRS-SR among young hoarders (mean = 19.7; sd = 4.9, range = 12–40) in the current study were also similar to scores reported in adults (mean = 21.1; sd = 3.9) [11]. However, a notable difference between hoarding symptoms in adolescence and adulthood is that they seem to affect females to a larger extent in the younger age group, a finding that also has been reported earlier [15]. An additional difference emerged when examining the frequency of excessive acquisition amongst individuals classed as having clinically significant hoarding difficulties. Approximately 30–40% of these individuals also reported at least moderate problems with acquisition, indicating that excessive acquisition may be less prominent in adolescents than it is in adults (about 85%; [44]). This lends further support to the inclusion of excessive acquisition as a diagnostic specifier rather than a core criterion in DSM−5. It is possible that excessive acquisition difficulties develop later in life when individuals with hoarding tendencies become financially independent and also have more control over their acquisitive behavior and space.

The obtained prevalence estimate of hoarding symptoms also indicates that these are at least as common as OCD during adolescence [45], [46], a finding that contradicts the notion that hoarding is merely is a subtype of OCD.

Given the lack of published data on the hoarding phenotype in adolescence and the potential of familial effects on clutter we also estimated a prevalence rate of 3.7% when excluding the requirement to have significant clutter in the participant’s room. It is still unknown whether the phenomenology of hoarding changes across the lifespan and whether the proposed diagnostic criteria for HD can be applied to adolescents without adaptation. Further exploration of the phenomenology of hoarding during adolescence involving face-to-face interviews and home visits will be needed in order to determine which features of hoarding are most prominent and problematic during this age period and whether the diagnostic criteria require adapting for their use in adolescents. Nevertheless, our estimates provide an important first step towards estimating the possible prevalence of hoarding difficulties in this age group.

Our findings also showed that OCD, to which HD has been linked traditionally, co-occurred in only 2.9% of adolescents who fulfilled hoarding criteria and at a similar rate among those who did not. This finding from a non-clinical sample suggests that the link between the two disorders might be especially tenuous during adolescence. Previous findings of high comorbidity between HD and ADHD in clinically ascertained samples of adults [12], [47] and youth [18] were not confirmed. We also found comparable rates of ASD in the hoarding and non-hoarding groups. Similarly, research in adults suggests that symptoms of autism are not more prevalent in subjects with HD compared to psychiatric controls [17]. Taken together, our findings suggest that in the majority of cases at the population level, hoarding symptoms are frequently present in the absence of other neurodevelopmental disorders, lending further support to the notion that HD might be a distinct nosological entity.

Finally, the twin analyses indicated that hoarding symptoms were moderately heritable in adolescence, although the magnitude of the genetic influence was much stronger in boys than in girls. This somewhat unexpected finding – hoarding symptoms are clearly heritable in adult men and women [10], [23] – was related to considerable shared environmental influences on hoarding symptoms amongst girls. However, similar findings of sex differences during adolescence have been reported previously regarding genetic and environmental influences for OCD, [48], [49] weight [50], and pubertal development [51]. Furthermore, dynamic developmental genetic effects from childhood to young adulthood, expressed in genetic attenuation and innovation (i.e. the decline in impact of certain genes and the activation of genes which previously had no effect) in symptoms of anxiety and depression are well documented [26]. It seems plausible that hoarding may become progressively more heritable over time as the influence of shared familial environment decreases when young people leave their parents’ home and have stronger control over their own living space. Longitudinal studies of the heritability of hoarding symptoms in young adulthood and beyond are needed to elucidate developmental trajectories of hoarding in both sexes.

The results should be interpreted considering several limitations. First, we based our classification of clinically significant hoarding symptoms on a measure not specifically validated in an adolescent population. Thus, the possibility that it did not capture hoarding symptoms equally well as in adults cannot be ruled out. Hopefully, the modification of the clutter item increased its relevance for this age group. Second, prevalence estimates should be seen as indicative rather than definitive because we could not conclusively rule out other medical or psychiatric conditions that are known to lead to hoarding behavior [52], [53]. Third, although ADHD, ASD and OCD are relatively stable prevalence-wise in the age span from 9/12 to 15 years, more precise estimates of their co-occurrence with hoarding symptoms would have been obtained had they been assessed at age 15 years and not at age 9/12; hence, we cannot totally exclude an under- or possibly overestimation of ADHD, ASD and OCD comorbidity. Fourth, albeit modeled closely after DSM-IV-TR-criteria [39], comorbid OCD was not determined using a validated measure, and was based solely on parental report. Thus, the OCD comorbidity rate might have been underestimated by parents and should therefore be interpreted cautiously. Fifth, and finally, since ASD and ADHD were significantly more common among non-responders, we cannot rule out that the true comorbidity of hoarding symptoms and neurodevelopmental disorders might be higher.

### Conclusion

This study is the first to investigate the occurrence of hoarding symptoms in a large population based sample of adolescents. Hoarding symptoms were prevalent among adolescents and usually appeared without co-occurring ADHD, ASD and OCD. Furthermore, the same etiological factors seemed to influence hoarding symptoms in both sexes although the genetic effect was much stronger in boys. Longitudinal studies are required to elucidate the developmental trajectories of hoarding symptoms and their heritability from adolescence onto adulthood.
